# Psychometric performance of the Arabic versions of the Functional Assessment of Cancer Therapy-Breast plus Arm morbidity (FACT-B + 4) in patients with breast cancer related lymphedema: cross-sectional study

**DOI:** 10.1186/s12905-022-01787-x

**Published:** 2022-06-03

**Authors:** Hind Abdulaziz Al-Hoqail, Mohammed T. A. Omar, Maha Mohammed Al-Marwani, Einas Soliman Al-Eisa

**Affiliations:** 1 Rehabilitation Health Sciences Department, College of Applied Medical Sciences, King Saud University, P.O. Box 145111, Riyadh, 4545 Saudi Arabia; 2 Physical Therapy Department, Faculty of Physical Therapy, Cairo University, Cairo, Egypt

**Keywords:** Quality of life, Breast cancer-related lymphedema, Psychometric properties, Reliability, Validity, FACT-B + 4

## Abstract

**Background:**

Burden of breast cancer it continues to increase largely because of the aging and growth of the world population and assessment of quality of life is an important outcome measure to facilitate and improved care among breast cancer survivors, the aim of this study was to evaluate evidence of reliability, validity, and responsiveness of the Arabic version of the FACT-B + 4 questionnaire among participants with breast cancer related lymphedema (BCRL) in Saudi Arabia.

**Methods:**

A prospective cross-sectional study, 51 participants with BCRL completed the Arabic version of FACT-B + 4. Internal consistency and test–retest-reliability were assessed using Cronbach’s alpha, intraclass correlation-coefficient (ICC), and limits of agreement according to the Bland Altman method, respectively. The validation studies were carried-out by examining predefined hypotheses (n = 14) for both construct and Known-groups validity. To investigate the responsiveness, the Arabic version of FACT-B + 4 questionnaire was administrated preoperative and 4 weeks postoperatively among the participants with breast cancer (n = 34).

**Results:**

The Cronbach alpha of the Arabic FACT-B + 4 total score was 0.90 and for the different subscales ranged from 0.74 to 0.89. Test–retest reliability for FACT-B + 4 total score and different subscales was found to be moderate to very strong (ICC 0.51–0.94). The Bland–Altman plot was adequate − 19.24 and 22.10 points. Measurement variability was acceptable for Arabic FACT-B + 4 and ARM subscale (standard error of measurement = 5.34, and 1.34). Moderate correlations (r = 0.42–0.62) were found between the subscale of the FACTB + 4 and the corresponding domains of SF-36. For known group validity, 72% (10 of 14) hypotheses on known group validity were accepted.

**Conclusion:**

FACT-B + 4 has adequate psychometric properties, thus making it useful for assessing QOL quality of life in Arabic speaking women with BCRL.

## Background

Breast cancer (BC) is the most common cancer affecting women worldwide [1], and in the Saudi Arabia [2]. Advancement in breast cancer treatment, such as surgeries and radiotherapy, have associated with an increased rate of long-term survival [3]. However, these interventions can cause damaging to the lymph nodes and/or vessels, leading to accumulation of protein-rich fluid, and development of breast cancer-related lymphedema (BCRL) [3–5].

The prevalence of BCRL varies in the literature and depends on treatment regimens [6], definition, methods of clinical detections and measurements techniques [7]. Recent review reported that one in five women who survive breast cancer would develop arm lymphedema [8]. In Saudi Arabia, BCRL has been a rising condition over the last 10 years, with an estimated incidence of 14.5% showing significance of this condition [9].

The most obvious symptom of BCRL is swelling of the affected limb, heaviness, tightness, and stiffness [10]. In addition, lymphedema can lead to physical impairment such as decrease strength, limited range of motion and fatigue, which can cause activity limitations and functional impairment in the affected arm, and negative body image, depression, and anxiety, all of which might be negatively influenced quality of life (QoL) [11–13]. Therefore, QoL is an important outcome measure to facilitate improved care of those with BCRL.

Several self-reported questionnaires used to detect the influence of BCRL on physical, functional, and social aspects of life among patients with BCRL [14–17], such as short form (SF-36) [14] and Disabilities of Arm, Shoulder, and Hand (DASH) [15], the European Organization for Research and Treatment of Cancer Quality of Life Questionnaire Core (EORTC QLQ‐C30) [17]. However, these instruments are generic, cancer specific, nonspecific, and not sensitive enough to detect the impact of BCRL on QoL. Therefore, using specific instruments is more likely to track changes in QoL [16].

One of the most used QoL questionnaires designed for patients with BCRL is the Functional Assessment of Cancer Therapy-Breast plus Arm morbidity (FACT-B + 4). The scale contains different subscales that address physical, social/family, emotional and functional wellbeing, with additional concerns related to breast cancer and an arm subscale [18]. FACT-B + 4 is a simple, short, self-administered questionnaire that was originally drafted and validated in the English language by Brady et al. in the USA, then the arm subscale (ARM) was developed and incorporated into the existing FACT-B by Coster et al. [18, 19]. Recently, the FACT-B + 4 questionnaire has been translated, culturally adapted, and validated to be used in different language and social environment [20–22].

Using a reliable and valid instrument to measure QoL, considering language and cultural differences, is crucial. To our knowledge, an Arabic version of the FACT-B + 4 is available, but evidence of reliability and validity of the Arabic version of the FACTB + 4 has not been established, and the psychometric properties of the FACTB + 4 also need to be estimated. Therefore, the aim of this study was to evaluate evidence of reliability, validity, and responsiveness of the Arabic version of the FACT-B + 4 questionnaire among participants with BCRL in Saudi Arabia.

## Methods

### Study design

A prospective cross-sectional study was carried out at oncology and physical therapy departments, King Saud Medical City, King Fahd Medical City (KFMC) and King Faisal specialized Hospital, Riyadh. Saudi Arabia. The institutional review boards and Ethics Committee of the entry hospitals approved this study.

### Participants

The study was conducted with 2 samples each participant signed informed consent form to participate and publish: (1) women with unilateral BCRL (inter-limb difference > 2 cm increase of any circumference), and (2) Saudi women with breast cancer survivors who were scheduled for surgery. Inclusion criteria were as following; age > 18 years, native Arabic speaking. Exclusion criteria were pregnant, active malignancy, and current infection/open wound, ongoing chemotherapy, and radiotherapy, local and/or systemic diseases causing impairment/disability in the affected limb, and cognitive impairment.

### Data collection procedures

The primary researcher who was Certified lymphedema therapist interviewed the eligible participants to gather sociodemographic and clinical data including age, educational level, and occupation, type of breast surgery, hand dominance, site, and duration of lymphedema, then checking the medical record to ensure the validity data. The BC participants were evaluated before surgery and after 1 month, while participants with BCRL were evaluated on two separate interviews (with 7 days interval). Details of the study procedures were given through verbal and written information, then each participant signed informed consent form. After that, participants completed independently battery of self-reported questionnaire included (1) Arabic version of FACT-B + 4 questionnaire and (2) SF-36.

Prior to administration of the FACT-B + 4 Arabic version, the instrument was piloted tested on twenty BCRL participants who met the inclusion criteria of the study. The participants completed the instrument and then during a face-to-face interview were asked to assess their understandability, the clarity of the scoring system, and completeness of the questionnaire [20 = 32]. The results indicated that the instructions and the structure of the FACT-B + 4 Arabic version instrument were understandable, clear, and easy; no one asks for clarification or explanation to help them respond to any of the items. However, forty percentage of the participants found item B4 (“I feel sexually attractive”) to be inappropriate and suggested that it be replaced with “I feel attractive (pretty)”.

### Instruments

#### Functional Assessment of Cancer Therapy-Breast plus Arm morbidity (FACT-B + 4)

The FACT-B + 4 is self-reported questionnaire designed to measure health related quality of life in participants diagnosed with BCRL and composed of 40 items divided into three parts: the general subscale on cancer (FACT-G), breast specific subscale (BCS) and ARM subscale [17]. The FACT-G divided into 4 subscales that measure physical well-being, social/ family well-being, emotional well- being, and functional well-being. The BCS has 10 items**,** while the ARM subscale has four items [18]. The Response system is 5 Likert scores varying between “0” shows “No at all” to “4” indicates ‘Very much”, where the positively stated items directly got scores from 0 to 4 points, and the negatively stated items are reversed [18, 19]. Permission to use the FACT-B + was obtained from the FACT organization (owned and copyrighted by David Cella).

#### Health related quality of life assessment using 36-Item Short-Form Health Survey (SF-36)

The SF-36 is a self-report generic measure of health status. The SF-36 is culturally adapted into Arabic and has a good reliability and validity within breast cancer survivors [23–25]. It comprises 36 items that are combined to form two main domains: physical and mental health status. Each subscale had score ranging from “0 to 100” scores where “0’ indicts worst health status and “100” represents optimal health conditions. The total score for each subscale computed according to the RAND 36 Health Survey manual and interpretation guide [26].

### Statistical analysis

The analyses were performed using IBM SPSS Statistics Version 26. Significance was set at *P* < 0.05. Descriptive statistics were computed for sociodemographic data and clinical characteristics.

### Reliability/floor/ceiling effects

The reliability analyses included internal consistency reliability of each subscale and total FACB + 4 using Cronbach’s alpha. Test–retest reliability was calculated using intraclass correlation coefficient (ICC) for absolute agreement (ICC2,1) and corresponding 95% confidence interval (CI) [27–29].

Wilcoxon test was conducted to examine systematic differences between the two interviews. Bland Altman plots were used to assess the extent of agreement between the two measures. Assuming that the differences follow normal distribution, the limits of agreement (LOAs) lie within d ± 1.96 × SD, where d represents the mean difference between the two measurements, and SD is the standard deviation of differences of each pair [30].

The standard error of measurement (SEM) was calculated from the following formulas: SEM = SD12√(1 − ICC) to evaluate the magnitude of the within-subjects variation, then the minimal detectable change at the 95% (MDC95%) was calculated based on SEM according to the following formulae MDC95 = 1.96 × SEM × √2 [27–29].

Potential ceiling and floor effects were measured by calculating the percentage of participants achieving the minimum or maximum scores. Ceiling and floor effects are considered being present if > 15% of the participants achieved the lowest and highest possible total score [31].

### Validity

Construct validity was evaluated in 2 ways. First, the correlation of FACT-B + 4 subscales (e.g. functional wellbeing, physical well-being, emotional well-being, and social/family well-being), with the corresponding domains of SF-36 including physical functioning, role-physical, role-emotional, and social functioning, respectively. Pearson correlation coefficient and the Spearman correlation coefficient were used for normally distributed scores and for the other scores, respectively. Second, the known-groups validity is used to discriminant between two groups that are already known to differ in terms of the variables of interest. In this study, known groups validity was examined by comparing scores of FACTB + 4 between the BC participants and BCRL Women were categorized with BCRL. The Mann–Whitney U test was used to compare the overall FACT B + 4 total and subscales scores between both groups. We formulated 14 hypotheses (Table 1) for both construct and known-groups validity based on literature [31]. Hypotheses were accepted when scoring a correlation coefficient 0.40. Construct validity was defined as very good if more than 90% of all 14 hypotheses were confirmed, good if 75% to 90% of the hypotheses were confirmed, and moderate if between 40 and 74% of the hypotheses were confirmed [29].

**Table 1:** Hypotheses to assess construct validity

Construct	Considering all correlation coefficients between most similarities’ subscale of the FACTB + 4 and the corresponding domains of SF-36, had moderate correlation 1. FACTB + 4 physical well-being and SF-36 physical function 2. FACTB + 4 physical well-being and SF-36 role-physical 3. FACTB + 4 emotional well-being and SF-36 role-emotional 4. FACTB + 4 social well-being and SF-36 social functioning
Known-groups validity	Patients with BCRL have: 5. A lower total score on the FACTB + 4 6. A lower score on teach subscale the FACTB than BC participants without lymphedema

*SF-36* Medical Outcomes Study 36-Item Health Survey, *FACTB + 4* Functional Assessment of Cancer Therapy-Breast plus Arm morbidity version4

Responsiveness was evaluated to examine the sensitivity of the scale to change over time in the BC group (n = 34) by comparing mean change scores of each subscale and total scores at 4 weeks postoperative minus baseline (before surgery) using Wilcoxon signed-ranks test. Based on Coster et al., recommendation, we postulated that within 4 weeks after surgery BC participants would have poor quality of life and higher levels of Arm morbidity than before surgery [18].

The ICCs, Cronbach alpha coefficients and correlation coefficients were interpreted as follows: 0 < 0.40 = weak; 0.40–0.74 = moderate; 0.75–0.90 = strong; and > 0.90 = very strong [28, 29, 32].

### Sample size estimation

G power software version 3.1.9.4 (University of Düsseldorf, Düsseldorf, Germany) was used for sample size calculation for the reliability analysis with the following parameters Alpha = 0.05, Power = 80%, and (r = 0.40). A minimum sample size of 46 is sufficient to detect a value of 0.40 for the ICC. An additional 10% drop-out rate was set thus sample size was increased to 51 participants [33]. While a sample size of 30 was considered the minimum required sample for examining test–retest reliability [32].

## Results

### Participants

Fifty-one participants with BCRL, and 34 BC participants without lymphedema involved in the present study. The two groups were comparable in age, educational level, body mass index, and types of breast surgery. All participants’ characteristics are presented in Table 2.

**Table 2:** Sociodemographic and clinical characteristics of the participants with BCRL (n = 51) and BC without lymphedema (n = 34)

Variables	BCRL group (n = 51)	BC-group (n = 34)
Age (mean ± SD) years	53.39 ± 10.89	56.73 ± 3.15*
Marital status		
Single	2 (3.9%)	3 (8.80%)^§^
Married	39 (76.50%)	26 (76.50%)
Divorced	7 (13.70%)	3 (8.80%)
Widowed	3 (5.90%)	2 (5.90%))
Education		
Elementary	21 (41.20%)	13 (38.24%) ^§^
Higher school	15 (29.40%)	13 (38.24%)
University degree	15 (29.40%)	8 (23.52%)
Family size		
< 3	5 (9.80%)	7 (20.60%) ^§^
3–5	15 (29.40%)	10 (29.40%)
> 5	31 (60.80%)	17 (50.00%)
Employment		
Housewife	36 (70.60%)	22 (64.70%) ^§^
Employment	3 (5.9%)	5 (14.70%)
Retried	12 (23.5%)	7 (20.60%)
BMI		
< 30 kg/cm^2^	15 (29.40%)	13 (38.20%) ^§^
> 30 kg/cm^2^	36 (70.60%)	21 (61.80%)
Breast surgery		
Mastectomy	31 (60.80%)	15 (44.10%) ^§^
Breast-conserving surgery	20 (39.20%)	19 (55.90%)
Lymphedema site		
Right	30 (58.8%)	–
Left	21 (41.20%)	–
Lymphedema duration		
< 3years	22 (43.10%)	-
3–5 years	17 (33.30%)	-
> 5 years	12 (23.50%)	-

*BC* breast cancer, *BCRL* breast cancer related lymphedema, *BMI* body mass index, *SD* standard deviation

*Non-significant difference (*P* > 0.05) Mann Whitney U-test

^§^Non-significant difference (*P* > 0.05) Chi square test

### Reliability/floor and ceiling

Table 3 shows the Cronbach alpha coefficients, ICCs, SEMs, and MDC95% for the Arabic FACT-B + 4 score, and each subscale scores in participants with BCRL.

**Table 3:** Cronbach alpha, Intraclass correlation coefficient, SEM and MDC and their corresponding 95% CI for the participants with BCRL

Subscales	Internal consistency α	FACT-B + 4 (Time 1)	FACT-B + 4 (Time 2)	*P* value	ICC	95% CI for ICC_2,1_	SEM	MDC_95_	95% CI for MDC_95_
PWB (7; 0–28)	0.84	19.70 ± 6.68	18.79 ± 5.66	0.50*	0.87	0.76–0.93	2.23	6.18	− 5.27 to 7.09
SWB (7; 0–28)	0.74	23.25 ± 5.38	23.12 ± 5.74	0.82*	0.79	0.62–0.89	2.54	7.06	− 6.65 to 7.47
EWB (6; 0–24)	0.75	21.05 ± 2.90	20.76 ± 2.83	0.23*	0.87	0.77–0.93	1.03	2.41	− 2.12 to 2.70
FWB (7; 0–28)	0.80	23.38 ± 3.75	23.32 ± 3.33	0.90*	0.72	0.67–0.85	1.87	5.20	− 5.14 to 5.26
BC 10 (10; 0–40)	0.75*	24.35 ± 6.61	23.94 ± 5.69	0.46*	0.86	0.74–0.93	2.31	6.39	− 5.98 to 6.80
ARM (5; 0–20)	0.84	10.85 ± 5.39	10.47 ± 5.58	0.23*	0.94	0.89–0.97	1.34	3.72	− 4.1 to 3.34
FACT-B TOI (24; 0–96)	0.85	67.44 ± 13.23	66.05 ± 11.18	0.42*	0.89	0.80–0.94	4.06	11.25	− 9.87 to 12.63
FACT-G (27; 0–108)	0.87	87.40 ± 14.17	86.00 ± 12.13	0.37*	0.92	0.84–0.96	3.73	10.34	− 8.94 to 11.74
FACT-B (37; 0–148)	0.89	111.75 ± 19.09	109.94 ± 16.48	0.32*	0.93	0.86–0.96	4.72	13.07	− 11.26 to 14.88
FACT-B + 4 (41; 0–164)	0.90	122.23 ± 23.02	120.79 ± 20.52	0.43*	0.94	0.88–0.97	5.34	14.80	− 13.37 to 16.23

Cronbach’s alpha excluding item (P2), *PWB* physical well-being, *SWB* social/ family well-being, *EEWB* emotional well-being, *FWB* functional well-being, FACT-B TOI = (PWB + FWB + BCS), FACT-B + 4 = PWB + SWB + EWB + FWB + BCS + ARM, *ICC* intraclass correlation coefficient, *CI* confidence interval, *SEM* standard error of the measurement, *MDC* minimal detectable change, *FACT-B + 4* Functional Assessment of Cancer Therapy-Breast plus Arm morbidity

*Non-significant *P* values (*P* > 0.05) for Wilcoxon test

The Cronbach α of the Arabic FACT-B + 4 total score was 0.90 and for the different subscales ranged from 0.74 (social/family well-being) to 0.89 (FACT-BC). For test–retest reliability; 34 BCRL participants completed the questionnaire for two interviews (baseline and within 7 days). The test–retest reliability of the Arabic FACT-B + 4 total score, ARM, FACT-BC, and FACT-G subscales score was very strong (ICCs > 0.90), that of PWB, SWB, EWB, BC, and FACT-TOI scores was strong (ICCs = 0.75–0.90), and moderate for FWB (ICCs = 0.51–0.72).

The total Arabic FACT-B + 4 score had a variability (SEM) of 5.34, between the two measurements. Furthermore, the MDC_95_ for the Arabic FACTB + 4 questionnaire total score was 14.80 points, with a decrease of the FACT-B + 4 score of 14 or more and an increase of 17 or more could be considered a clinically relevant change. The ARM subscale score had a variability (SEM) of 1.34, between the two measurements. Furthermore, The MDC_95_ was 3.72, with a decrease of the ARM score of 4 or more and an increase of 4 or more could be considered a clinically relevant change.

**Fig. 1:**
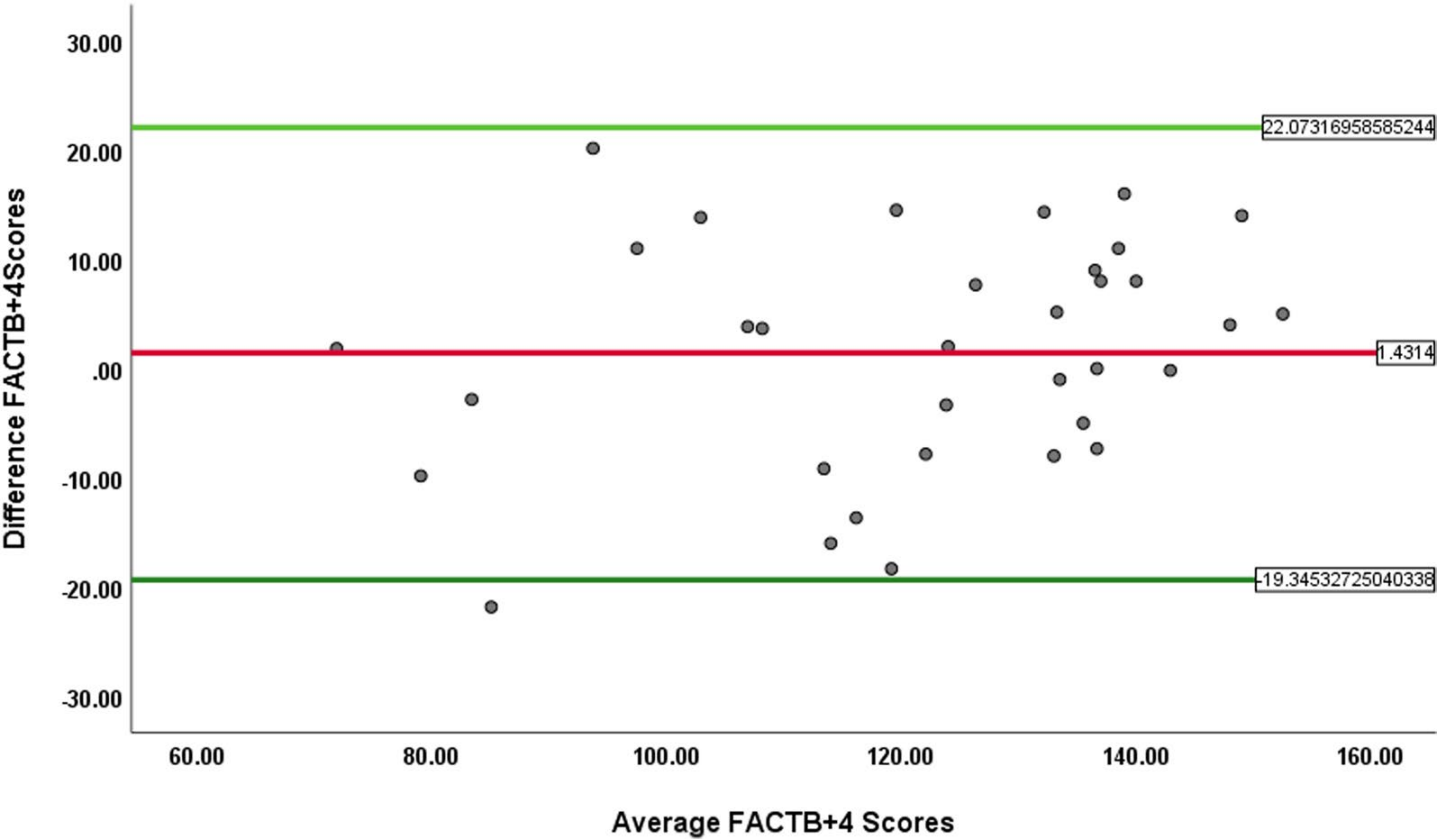
Bland–Altman plot for the total score of the Arabic FACTB + 4 questionnaire

As shown in the Table 3, the Wilcoxon signed-rank test for total score of the Arabic FACTB + 4 and each subscale score did not show any significant differences (*P* < 0.05) between both test occasions. Figure 1 shows the Bland–Altman graph of the FACT-B + 4 questionnaire. The value of mean differences was 1.4314 (SD, ± 10.54.), and the limits of agreement for the total scores were -19.24 and 22.10 points This indicated that, questionnaire had a good random distribution around zero, with few points out of range.

The floor/ceiling effects revealed that the percentages of participants scoring at the floor/ceiling level for total scores of the Arabic FACT-B + and subscales were less than 15%; however, we found a ceiling effect of 19.6% and 15.7% for the SWB and EWB, respectively.

### Validity

Table 4 presented the associations between most similarities’ subscale of the FACTB + 4 and the corresponding domains of SF-36. The analysis of associations between PWB, SWB and EWB, FWB dimensions of the Arabic FACTB + 4 and the corresponding dimensions on SF-36; role limitation due to physical function, social functioning, role limitation due to emotional problems and physical function dimension revealed moderate correlation (r = 0.42–0.62), therefore, all 4 hypotheses were accepted.

**Table 4:** Correlation between the Arabic FACTB + 4 and other SF-36

Subscales	PWB	SWB	EWB	FWB
Physical function	–	–	–	0.54*
Role limitation due to physical health	0.60*	–	–	
Role limitation due to emotional problems	–	–	0.60*	–
Social functioning	–	0.42*	–	–

*PWB* physical well-being, *SWB* social/family well-being, *EEWB* emotional well-being, *FWB* functional well-being

*Correlations significant at the .01 level

Table 5 represents known group validity of the Arabic FACTB + 4. The total score on Arabic FACTB + 4 and the scores FACT-B, FACT-TOI, ARM, the BC subscales were significantly higher (*P* < 0.001) for BC participants without lymphedema than for those with BCRL, Participant with and without lymphedema had a comparable score on the PWB, EWB, FWB, and FACT-G. Analysis of Aram subscale items revealed that BCRL participants significantly had more arm problems than BC without lymphedema, including swelling (96.08% vs 12.19; *P* = 0.001), pain (74.51% vs 24.39%; *P* = 0.01), arm movement (66.67% vs 17.07%; *P* = 0.01), numbness (66.67% vs 19.51%; *P* = 0.01), and stiffness (51% vs 4.87; *P* = 0.01), therefore, 6 out of 10 hypotheses were accepted. Construct validity of the Arabic FACTB + 4 was moderate, as 72% (10 of 14) of the hypotheses were established.

**Table 5:** Mean scores of the Arabic version of FACTB + 4 for participants with BCRL and breast cancer without lymphedema

Subscales	Mean (SD)		MD (95%CI)	*P* values
	BCRL (n = 34)	BC without lymphedema (n = 34)		
PWB (7; 0–28)	19.70 ± 6.68	19.91 ± 5.75	− .20 (− 3.22 to 2.810	0.89*
SWB (7; 0–28)	23.25 ± 5.38	26.25 ± 2.84^ǂǂ^	− .20 (− 3.22 to 2.81)	0.006
EWB (6; 0–24)	21.05 ± 2.90	21.91 ± 2.86*	.14 (− 1.25 to 1.55)	0.83*
FWB (7; 0–28)	23.38 ± 3.75	23.94 ± 3.69*	− .55 (− 2.26 to 1.15)	0.51*
BC 10 (10; 0–40)	24.35 ± 6.61	29.44 ± 5.16^ǂǂ^	− 5.08 (− 7.96 to − 2.20)	0.001
ARM (5; 0–20)	10.85 ± 5.39	17.35 ± 4.01^ǂǂ^	− 6.88 (− 9.2 to − 4.54)	0.001
FACT-B TOI (24; 0–96)	67.44 ± 13.23	73.29 ± 10.58^ǂ^	− 5.85 (− 11.65 to .05)	0.04
FACT-G (27; 0–108)	87.40 ± 14.17	91.01 ± 9.41*	− 3.60 (− 9.43 to 2.21)	0.22*
FACT-B (37; 0–148)	111.75 ± 19.09	120.45 ± 12.55^ǂ^	− 8.69 (− 16.52 to − .87)	0.03^ǂ^
FACT-B + 4 (41; 0–164)	122.23 ± 23.02	137.80 ± 13.83^ǂǂ^	− 15.57 (− 24.77 to − 6.38)	0.001

*PWB* physical well-being, *SWB* social/family well-being, *EEWB* emotional well-being, *FWB* functional well-being, FACT-B TOI = (PWB + FWB + BCS), FACT-B + 4 = PWB + SWB + EWB + FWB + BCS + ARM, *FACT-B + 4* Functional Assessment of Cancer Therapy-Breast plus Arm morbidity

*Non-significant *P* values (*P* > 0.05) for Mann–Whitney U test

^ǂ^Significant *P* values (*P* < 0.05) for Mann–Whitney U test

^ǂ ǂ^Significant *P* values (*P* < 0.01) for Mann–Whitney U test

Table 6 represents the responsiveness to change of FACT-B + 4 scores in BC participants (n = 21) before and after surgery. A significant declined (*P* < 0.01) was reported in total FACTB + 4 score and in all subscales (*P* < 0.05) except for EWB and BC.

**Table 6:** Responsiveness to change of FACT-B + 4 scores in BC participants before and after surgery

Subscales	Pre-operative Mean ± SD	4 weeks post-operative Mean ± SD	MD (95% CI)	*P* values
PWB (7; 0–28)	20.85 ± 5.83	17.38 ± 5.45^ǂ^	3.47 (0.05–6.89)	0.04
SWB (7; 0–28)	26.18 ± 2.76	24.12 ± 3.87^ǂ^	2.07 (0.18–3.97)	0.03
EWB (6; 0–24)	20.57 ± 2.69	20.85 ± 2.92*	− .28 (− 2.19 to 1.62)	0.75
FWB (7; 0–28)	23.52 ± 4.53	20.67 ± 3.76^ǂ^	2.84 (0.72–4.97)	0.01
BC 10 (10; 0–40)	29.04 ± 5.86	27.90 ± 4.57*	1.14 (− 2.08 to 4.37)	0.46
ARM (5; 0–20)	19.04 ± 2.13	14.46 ± 4.968^ǂǂ^	4.58 (2.01–7.14)	0.001
FACT-B TOI (24; 0–96)	73.42 ± 12.37	65.966 ± 10.53^ǂ^	7.46 (0.47–14.46)	0.03
FACT-G (27; 0–108)	91.13 ± 10.81	83.01 ± 11.44^ǂ^	8.12(1.57–14.69)	0.01
FACT-B (37; 0–148)	120.18 ± 14.95	110.92 ± 14.40^ǂ^	9.26 (0.74–17.78)	0.03
FACT-B + 4 (41; 0–164)	139.23 ± 15.68	125.36 ± 16.98^ǂǂ^	13.84 (4.14–23.54)	0.007

*PWB* physical well-being, *SWB* social/family well-being, *EEWB* emotional well-being, *FWB* functional well-being, FACT-B TOI = (PWB + FWB + BCS), FACT-B + 4 = PWB + SWB + EWB + FWB + BCS + ARM, *FACT-B + 4* functional assessment of cancer

^ǂ^Significant *P* values (*P* < 0.05) for Wilcoxon test

^ǂ ǂ^Significant *P* values (*P* < 0.01) for Wilcoxon test

*Non-significant *P* values (*P* > 0.05) for Wilcoxon test

## Discussion

The lack of validated self-reported Arabic outcome measures in patients with breast cancer related lymphedema has restricted research. Therefore, the aim of this study was to evaluate evidence of psychometric performance of the Arabic version of the FACT-B + 4 questionnaire among participants with BCRL in Saudi Arabia.

Piloted tested showed clarity, acceptability, and understandability of the Arabic FACTB + 4 version with no difficulty regarding scale’s instructions and scoring system. There are no floor and ceiling issues reported during using the Arabic FACT-B + 4. These findings supporting the content, relevance, and comprehensibility of Arabic FACTB + 4 version with the original version developed by Coster et al. [18] and show its suitable as specific outcome measures to be used in assessments of Arabian women with a BCRL both in research and in clinical settings.

The Cronbach alpha of the FACTB + 4 in the Arabic-speaking BCRL patients (α = 0.90) was somewhat higher than, but comparable with, the estimates of internal consistency (0.88) for both the original English version [18], and Brazilian versions [31]. The SWB, EWB, FWB, and ARM subscale had reliability estimate of (0.74–0.89) that was comparable with the original English version (0.76–0.88) [18], and Brazilian versions (0.66–0.84) [31] and Spanish version (0.69–0.89) [20]. The Cronbach alpha of the PWB (α = 0.84) and BC (α = 0.75) was somewhat higher than the estimates of internal consistency for both the original English version (0.71 and 0.62) [18], Brazilian versions (0.75 and 0.66) [31], and Spanish version (0.75 and 0.52) [20]. Similar to current results, Italian version FACTB + 4 reported adequate Cronbach’s alpha for PWB (0.810 and BC (0.74) [21].

The ICCs of the Arabic version of FACT-B + 4 total scores and the scores on each subscale varied between moderate and very strong (ICC = 0.72–0.94). These results are in line with the early finding of Coster et al. [18], who reported significant ICC value for an English version of FACT-B + 4 (ICC = 0.97) and the Brazilian version 0.86 (ICC = 0.86, 95% CI 00.80 to 0.90) in a group of BCRL (n = 18) [22, 31]. The ICCs of the Arm subscale was very strong (ICC = 0.94, 95% CI 0.89–0.97) and somewhat lower than, but comparable with that reported by English version (ICC = 0.97, 95% CI 0.79–0.95) [18]. However, Brazilin [31] and Spanish [20] versions reported lower ICC for Arm subscale (0.75–0.88), respectively. The reliability estimate of the Arabic BC subscale was very strong (ICC = 0.92, 95% CI 0.84–0.96) while Oliveria et al. [31] and Martinez et al. [20] mentioned lower test–retest reliability for BC subscale (ICC 0.75 and 0.82), respectively.

The average values of FACTB + 4 total score and each subscale in the test and retest showed that participant with BCRL had similar scores in the Arabic FACT-B + 4 with different administrations time.

The Arabic FACT-B + 4-measurement error was quantified in the current study using SEM and MDC. The reported MDC indicates that the score in the Arabic FACT-B + 4 needs to change by at least 14.80 points for total scales and 1.34 points for Arm subscales, in order to describe that change in the score as a true change in the participants with BCRL. However, none of these studies examined minimal detectable changes, as recommended by Lexell and Downham [28]. The measurement error by SEM for the Arabic of version FACT-B + 4 total score, and each subscale had somewhat smaller SEMs in comparison to both original English questionnaire [18] and PWB, EWB, FWB, BC, and Arm subscales and FACT-B + 4 total score for Brazilian version [31].

There are several differences in conditions for examining reliability which could influence the test results. For test–retest analyses, various time intervals were selected between test–retest periods. In the current studies, the time interval for test–retest analyses was 1 week based on the original English version [18], and Spanish version [20], while in the Italian version, it was 2 days among participant underwent breast cancer surgery [21] and 30 days for the Brazilian version [31]. All patients completed the Brazilian version under supervision at both test and retest, whereas the Arabic questionnaires was completed without supervision since the FACT-B + 4 is self-administered. In addition, the variability in samples used such as participant with recent breast surgery in Italian version [21], mixed group of BCs with and without lymphedema [31]. These reflected non-homogeneity in response to scale rather than equivalence problem of the Arabic version.

Construct validity was tested in 2 ways and gave good results in the patients with BCRL. The PWB, SWB, EWB and PWB scores of the Arabic FACT-B + 4, had moderate correlation (between 0.42 and 0.62), with the expected domains of the SF-36. Other studies found comparable [20] or slightly lower correlations [31], between their questionnaire and a questionnaire already tested on validity. The FACT-B + 4 showed correlations between 0.31 and 0.41 with similar domains in the SF-36. On the other hand, World Health Organization Quality of Life-brief (WHOQOL-brief), had good correlation with FACTB + 4; physical health domain of the WHOQOL-bref and PWB scale (r = 0.69), social relationships domain of the WHOQOL-bref and SWB scale of (r = 0.62); psychological domain of the WHOQOL-bref and EWBT (r = 0.61) [31]. Furthermore, these results, comparable with Martınez et al., reported a significant correlation of the SF-36 questionnaire with FACT-B + 4 except for vitality and social function [20]. However, in a study by Coster et al., this analysis was not performed [18].

Concerning the second method of the construct validity analyses, participants with BCRL had a significantly lower total score on the Arabic version FACT-B + 4, lower scores on the other subscales, including ARM subscale. These results indicated that the arm subscale, the breast cancer concerns scale, and the FACT-B + 4 were able to discriminate between participants with arm morbidity and those without arm problems. This finding confirmed the results of Coster et al. [18] who found significant differences in mean scores between participants suffering from lymphedema and without lymphedema.

This study found that breast cancer patients reported a significant decline QOL in terms of the physical, functional, social function and ARM subscales as well as FACT-G, FACT-TOI, FACT B and FACTB + 4 total FACT-B + at 4 weeks after surgery in comparison to preoperative status. These findings were similar to the result from in Coster et al.’s [18] study, which suggests that patients will suffer from arm morbidity more than 1 month after surgery. These findings suggested that the arm subscale and the Arabic FACT-B + 4 were sensitive to changes in arm morbidity during the postoperative period.

A strength of the current study was that different aspects of reliability, validity and responsiveness of the FACT-B + 4 were investigated and followed the recommended methods and preferred statistical analyses outlined by the COSMIN group [34]. Our study had some limitations. The inclusion criteria included the largest possible number of women with breast cancer-related lymphedema, regardless of their severity and stage. The wide variety in the type of surgery and time since surgery may have become a limitation because a more homogeneous sample regarding lymphedema stage and severity or surgery types couldn’t have similar changes in QoL. Among the limitations of this study, the sample of patients with BCRL comes from a central province of Saudi Arabia, which limits the generalizability of data because of cultural differences between Saudi Arabia’s provinces. However, we believe this will have a minimal effect on the generalizability of the results, because the FACT-B + 4 using Modern Standard Arabic, the language used in books, newspapers, magazines, media, formal speech, and communications and the most common form of Arabic taught in primary education [35]. Despite achievement of adequate acceptance of validity hypothesis (10 of the 14; 72%). Future study should emphasis to improve the remaining items that could not be shown to be effective.

## Conclusion

The Arabic FACT-B + 4 version showed strong internal consistency, test–retest reliability, and moderate construct validity similar to the original questionnaire. These results may enable the Arabic FACT-B + 4 version to be used to assess quality of life in Arabic speaking women with BCRL.

## Data Availability

The datasets used and analyzed during the current study are available from the corresponding author on reasonable request.

## Abbreviations

AAOS: American Association of Orthopedic Surgeons
ARM: Arm subscale
BC: Breast cancer
BCS: Breast cancer subscale
BCRL: Breast cancer-related lymphedema
EWB: Emotional well-being
FACT-G or FACT: Functional Assessment of Cancer Therapy FACT-G or FACT
FACT-B: Functional Assessment of Cancer Therapy-Breast
FACT-B+: Functional Assessment of Cancer Therapy-Breast plus Arm morbidity
FWB: Functional well-being
HRQOL: Health-related quality of life
SF-36: Item Short Form Health Survey-36
MAPIRIM: MAPI research institute methodology
MMST: Mini–mental state examination
PWB: Physical well-being
QOL: Quality of life
SWB: Social/family well-being
TOI: Trial outcome index
WHO: World Health Organization
